# Application of bedside real-time ultrasound–guided umbilical venous catheterization in neonates and development of an XGBoost-based complication prediction model: a retrospective study

**DOI:** 10.3389/fped.2026.1797779

**Published:** 2026-05-21

**Authors:** Qiu-Ping Huang, Mei-Xiang Zou, Miao-Chan Zhu, Xiu-Cong Mo, Guina Pan, Jing Wu

**Affiliations:** 1 Department of Neonatology, Panyu Maternal and Child Care Service Centre of Guangzhou, Guangzhou, Guangdong, China; 2 Department of Ultrasound, Panyu Maternal and Child Care Service Centre of Guangzhou, Guangzhou, Guangdong, China

**Keywords:** beside beal-time ultrasound, complication, neonate, risk factors, umbilical vein catheterization

## Abstract

**Purpose:**

To assess the application value of bedside real-time ultrasound (US) for umbilical venous catheterization (UVC) in neonates and evaluate its safety and predictive factors for complications.

**Study design and methods:**

This was a single-arm, retrospective, observational study conducted at a single institution, without a direct internal control group (such as X-ray guided placement) for comparison. A total of 132 neonates who received UVCs using real-time US guidance with color Doppler were included. Clinical variables, including success rates and complications within 2 weeks post-insertion, were captured. Factors contributing to complications were identified using Multivariate Logistic Regression. An XGBoost model and SHAP analysis were employed to predict and interpret complication risks.

**Results:**

The success rate for one-time catheter insertion was 95.45%, the mean indwelling time was 7.31 ± 0.27 days, and the overall complication rate was 15.15%, with accidental catheter dislodgement occurring in 9.09% of cases. Independent risk factors for complications included non-standard catheterization, preterm birth, lower birth weight, and a lower 1 min Apgar score. The XGBoost model performed well in predicting complications, with SHAP analysis identifying non-standard technique and birth weight as predominant influences.

**Conclusion:**

Bedside real-time US is shown to be a safe and accurate procedure for UVC placement in neonates. While this study did not include a direct comparison group, the high success and low complication rates align with literature suggesting that US can improve accuracy and potentially decrease radiation exposure compared to routine X-ray verification. The observed improvements are inferred through comparisons with external literature; therefore, further randomized controlled trials are needed to quantify its superiority over X-ray guidance.

## 1 Introduction

Umbilical venous catheterization (UVC) is a common procedure in the NICU, especially in premature infants. It provides rapid and reliable central venous access for fluid administration, medication delivery, blood sampling, and transfusions. Compared to peripheral venous cannulation, UVC offers several advantages. It is particularly useful in low-birth-weight and critically ill neonates, whose peripheral veins are often fragile, poorly visible, or difficult to cannulate (1–3). However, UVC placement is a technically demanding procedure and is associated with both insertion-related and catheter-related complications.

Historically, UVC placement has relied on external anatomical landmarks, with catheter position confirmed by radiography after insertion. Both approaches are highly dependent on individual anatomical variation. Moreover, the delay between placement and the detection of malposition often necessitates repeated exposure to ionizing radiation (4). Malposition of the catheter tip can lead to mechanical injury, vascular or organ damage, and other catheter-related complications. In recent years, real-time bedside ultrasound has emerged as an alternative technique that allows direct visualization of vascular anatomy and catheter advancement. It enables real-time assessment of catheter direction, insertion depth, and tip location, thereby improving accuracy and eliminating radiation exposure during placement (5). Ultrasound-assisted UVC has been shown to reduce the incidence of catheter malposition and allows for immediate correction during insertion.

Despite its advantages, real-world evidence on ultrasound-assisted UVC placement remains limited, and few studies have systematically evaluated the complications associated with this technique in routine clinical settings. Moreover, there is a lack of research applying predictive modeling approaches to identify neonates at elevated risk for UVC-related complications—an important step toward enabling individualized monitoring and management. Retrospective data from single centers in China offer valuable empirical insights, reflecting real-world clinical practice beyond the confines of tightly controlled trial environments and complementing existing randomized clinical trials.

Accordingly, this study aims to evaluate the clinical value of bedside ultrasound for UVC insertion in neonates by identifying factors associated with complications and developing a predictive model using XGBoost with SHAP interpretation to assess complication risk.

## 2 Methods

### 2.1 Study design, subjects, and ethics

This was a single-arm, retrospective, observational study conducted at a single center. Therefore, the findings reflect the specific clinical environment and resources of our institution, which may limit their generalizability. The study protocol was approved by the Institutional Review Board (IRB) in accordance with the Declaration of Helsinki. Patient confidentiality was maintained throughout the study.

**Figure 1:**
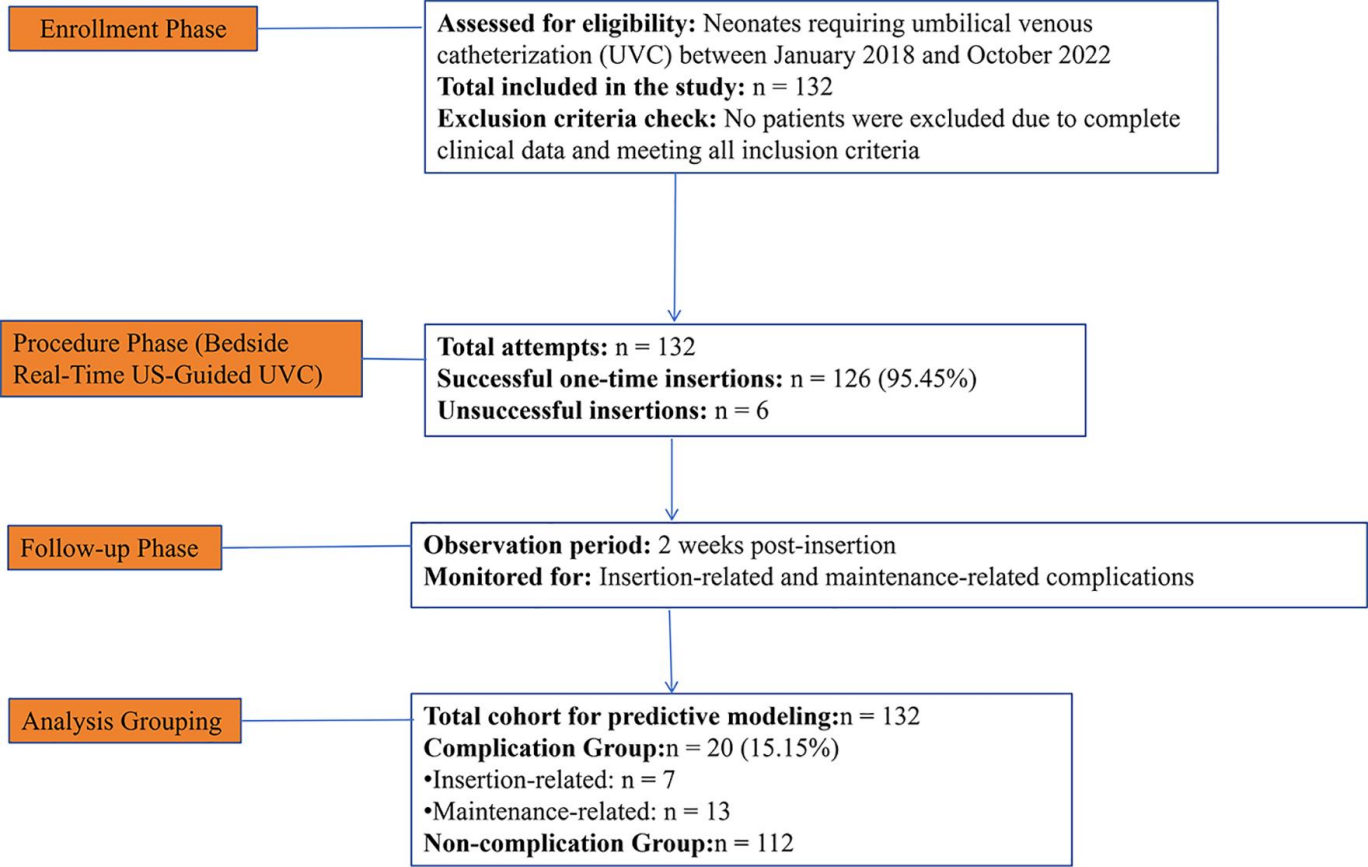
Study flow diagram of participant enrollment and progression. This flowchart details the screening process, the exclusion of 0 neonates, the inclusion of the 132 neonates who met all criteria, and the clinical outcomes including successful (*n* = 126) and unsuccessful (*n* = 6) insertion attempts.

All neonates who met the inclusion criteria during the enrollment period were included in the study. Between January 2018 and October 2022, a total of 132 neonates requiring UVC were screened, all of whom met the eligibility criteria; none were excluded. The sample size was determined by the consecutive enrollment of all eligible patients during the study period, aiming to maximize statistical power and ensure representativeness of routine clinical practice. Ultimately, 132 neonates were enrolled and included in the final analysis. A study flow diagram detailing the enrollment and progression of patients is presented in Figure 1.

All UVC procedures were performed at the bedside under color Doppler ultrasound guidance. The mean time from birth to admission was 52.12 ± 5.69 min. The cohort included 69 male and 63 female infants, with a mean birth weight of 1.69 ± 0.24 kg. The most common underlying conditions were respiratory distress syndrome (*n* = 119), hyperbilirubinemia (*n* = 6), congenital heart disease (*n* = 5), and intraventricular hemorrhage (*n* = 2); additionally, 103 infants (78.0%) required ventilator support and 4 (3.0%) required inotrope use. The mean Apgar score at one minute postpartum was 8.5 ± 1.1.

The study population inclusion criteria included: (1) gestational age at birth of less than 37 weeks; (2) clinical indication for UVC with anticipated need for intravenous infusion, or IV medications, for greater than two (2) weeks; (3) postnatal age of between 0 and 2 days on the date of UVC catheter insertion; (4) anticipated duration of catheter indwelling between 1 and 11 days; and (5) complete patient clinical data available. Exclusion criteria were as follows: (1) contraindications to UVC such as omphalitis, omphalocele, necrotizing enterocolitis, peritonitis, active bleeding, or circulatory disorders of the lower limbs or buttocks; and (2) incomplete clinical records.

### 2.2 Bedside real-time ultrasound-guided UVC technique

All ultrasound-guided UVC procedures were performed at the bedside by a dedicated team of senior neonatologists, each with at least five years of NICU experience. Prior to the study, all operators completed a standardized certification program in point-of-care ultrasound (POCUS) and had performed a minimum of 30 supervised ultrasound-guided UVC insertions. This ensured a high level of technical proficiency and consistency, particularly in identifying the junction of the inferior vena cava (IVC) and the right atrium (RA).

A portable color Doppler ultrasound system equipped with a high-frequency linear transducer (10–14 MHz) was used to provide the axial resolution required for neonatal vascular imaging. To maintain consistency across different ultrasound platforms, key image parameters—including gain, depth, and focal zone—were standardized and then individually optimized for each infant to ensure clear visualization of the vessel wall and catheter tip.

Following standard skin disinfection and sterile draping of the umbilical region, a single-lumen umbilical catheter (3.5 Fr or 5 Fr, selected based on birth weight) was inserted to an estimated depth according to the infant's body weight. Real-time ultrasound was used throughout the advancement of the catheter, allowing continuous visualization of the umbilical vein and assessment of catheter depth and direction in both longitudinal and transverse planes (Supplementary Figure S1).

Ultrasound was used for both initial verification and any subsequent repositioning. Routine post-insertion X-ray was not performed for all infants; radiographs were obtained only when ultrasound visualization was inadequate or when complications were clinically suspected. Therefore, a systematic correlation between ultrasound and X-ray findings across the entire cohort was not feasible.

Once correct positioning was confirmed, the catheter was secured using sterile adhesive tapes and a transparent semi-permeable dressing to minimize dislodgement and allow ongoing site inspection. Care was taken throughout the procedure to prevent air embolism. Catheter patency was maintained with a continuous infusion of total parenteral nutrition (TPN) or crystalloid solutions containing low-dose heparin (0.5–1.0 U/mL), following an initial flush with heparinized saline.

### 2.3 Definition and classification of complications

Complication data were collected from clinical records and imaging findings over a two-week period following UVC insertion. This observation window was selected to capture acute and subacute events directly related to the insertion procedure or early catheter maintenance. An event was attributed to UVC if it occurred within this timeframe and had a plausible connection to either the insertion or the indwelling of the catheter.

Complications were categorized into two types: insertion-related and catheter-related (maintenance-related). Insertion-related complications were defined as those directly resulting from the catheterization procedure. “Non-standard catheterization” was specifically defined as any procedure requiring more than two repositioning attempts, inability to definitively visualize the catheter tip at the IVC-RA junction during placement, or any technical deviation from the standardized ultrasound-guided protocol described in Section 2.2. Such complications included catheter malposition, failure to correctly identify the tip at insertion, early dislodgement, and bleeding or hematoma at the umbilical site, as determined by clinical assessment and available imaging.

Iatrogenic complications were those arising from the continued presence of the indwelling catheter. These included local signs of inflammation such as redness, swelling, or exudate at the umbilical site. Catheter-related infections were defined based on clinical documentation of suspected infection associated with the indwelling line. In the absence of an alternative diagnosis, any complication developing after catheter placement was also considered potentially related.

All complications were independently reviewed and classified by senior neonatologists, with ultrasound or radiographic evidence used when available.

### 2.4 Observation indexes

The following clinical parameters were collected: one-time catheterization success rate, catheter placement time, number of fixation attempts, catheter indwelling duration, episodes of accidental (unplanned) catheter dislodgement, and the timing of any UVC-related complications. In addition, data on potential confounding factors—including ventilator support, inotrope use, and procedural context (planned vs. emergency insertion)—were also recorded.

### 2.5 Statistical analysis and prediction modeling

All statistical analyses were performed using SPSS version 22.0. Categorical variables were expressed as frequencies and percentages and compared using the chi-square test. Continuous variables were presented as means with standard deviations and compared using independent-samples *t*-tests. Variables that showed statistical significance in univariate analyses were subsequently entered into a multivariate logistic regression model to identify independent risk factors for UVC-related complications, with adjustment for potential confounders such as clinical acuity (e.g., ventilator support or inotrope use) and operator-related factors.

To mitigate the risk of overfitting, clinically relevant variables were selected *a priori* based on expert opinion prior to model construction. Given the exploratory nature of the study, the sample size was deemed adequate for developing a machine learning-based prediction model. The XGBoost algorithm was applied to predict the risk of UVC-related complications. To ensure model stability given the limited number of events (*n* = 20), internal validation was performed using calibration and resampling techniques.

Model performance was evaluated using discrimination metrics, including the area under the receiver operating characteristic curve (AUC), along with calibration curves and clinical decision curve analysis. SHapley Additive exPlanations (SHAP) were employed to enhance model interpretability by quantifying each predictor's contribution to individual risk estimates. A two-tailed *p*-value < 0.05 was considered statistically significant.

## 3 Results

### 3.1 Outcomes of bedside ultrasound guided umbilical catheterization

Among the 132 neonates, real-time bedside ultrasound-guided UVC was successful in 126 cases, yielding a 95.45% success rate with a single catheterization attempt (Figure 1). There were six unsuccessful attempts at catheterisation. On average, catheters were in place for 7.31 ± 0.27 before accidental dislodgement in 12 cases (9.09%).

Catheters placed in a non-ideal position or that experienced early dislodgement resulting in the need for removal were classified as insertion-related procedural events in seven cases. No statistically significant differences were found in the rates of one-time catheterization success, overall complication rate, or accidental catheter dislodgement between very low birthweight and low birthweight neonates (Table 1).

**Table 1:** Comparison of the success rate of one-time catheterization, complication rate, and accidental catheter dislodgement rate between very low birth weight infants and low birth weight infants.

Groups	*n*	Complication rate	Success rate of one-time catheterization	Accidental catheter dislodgement rate
Low birth weight infants	25	2 (8.00)	24 (96.05)	1 (4.00)
Very low birth weight infants	107	18 (16.82)	102 (95.33)	11 (10.28)
*χ* ^2^		0.637	0.150	0.357
*p*		0.425	0.698	0.550

Comparisons between very low birth weight infants and low birth weight infants were performed using the *χ*^2^ test. A *p-value* < 0.05 was considered statistically significant. Very low birth weight was defined as birth weight <1.50 kg, and low birth weight as 1.50–2.49 kg.

### 3.2 Complications associated with UVC

During the first two weeks following catheter placement, a total of 20 UVC-related complications (15.15%) were identified and classified as either insertion-related or catheter-related (maintenance-related) according to predefined criteria.

Insertion-related complications included catheter removal due to suboptimal initial placement or early dislodgement (*n* = 7), signs suggestive of portal or intrahepatic pneumatosis occurring within hours of insertion (*n* = 2), and blood reflux associated with catheter positioning (*n* = 2).

Maintenance-related complications consisted of abdominal skin redness or swelling at the umbilical site (*n* = 4), drainage from the insertion site (*n* = 2), abdominal distension with suspected progression to abdominal wall pneumatosis (*n* = 2), and chyle leakage (*n* = 1).

Notably, no severe complications—such as cardiac effusion, cardiac rupture, clinically significant arrhythmias, catheter-related bloodstream infections, or mortality—were observed in this cohort.

### 3.3 Univariate analysis of factors associated with UVC complications

Univariate analysis revealed no statistically significant associations between UVC-related complications and infant sex, mode of delivery (vaginal vs. cesarean section), or Apgar scores at 5 and 10 min (all *p* > 0.05). In contrast, the following factors were significantly associated with the occurrence of complications: catheterization insertion time, non-standard catheterization, indwelling time >8 days, preterm birth, number of fixation attempts, birth weight below gestational age, and lower 1 min Apgar score (all *p* < 0.05) (Table 2).

**Table 2:** Univariate analysis of factors associated with complications of umbilical vein catheterization.

Items	Complication group	Non-complication group	*t*/*χ*^2^	*p*
N	20	112		
Time from birth to admission (min)	50.33 ± 4.23	47.62 ± 3.98	0.951	0.111
Gender (male)	9	60	1.692	0.651
Cesarean section (case)	19	110	1.234	0.212
Number of intubation fixations (times)	1.11 ± 0.22	1.46 ± 0.15	0.856	0.032
Birth weight (kg)	1.61 ± 0.22	1.78 ± 0.14	1.362	0.048
Intubation time (min)	8.12 ± 2.33	10.21 ± 1.58	6.325	0.000
Non-standard catheterization (case)	6	5	9.521	0.000
Catheter indwelling time (d)	11.32 ± 1.05	8.74 ± 1.29	5.284	0.047
Premature delivery (cases)	20	52	9.321	0.000
Neonatal Apgar score (1 min)	7.8 ± 0.13	9.1 ± 0.11	3.547	0.014
Neonatal Apgar score (5 min)	9.4 ± 0.14	9.5 ± 0.10	1.362	0.058
Neonatal Apgar score (10 min)	9.8 ± 0.1	9.7 ± 0.1	0.254	0.124
Ventilator support (case)	14	89	0.275	0.600
Inotrope use (case)	0	4	—	0.486

The independent-samples *t*-test was used to compare continuous variables, and the χ^2^ test was used to compare categorical variables. *p-*values < 0.05 were considered statistically significant. Complications are adverse events related to insertion or maintenance that occur during umbilical venous catheterization or during follow-up.

Ventilator support and inotrope use were also evaluated in univariate analysis (*p* = 0.600 and *p* = 0.486, respectively) and were subsequently included in the multivariate logistic regression model as adjustment variables for clinical severity.

### 3.4 Multivariable logistic regression analysis

**Figure 2:**
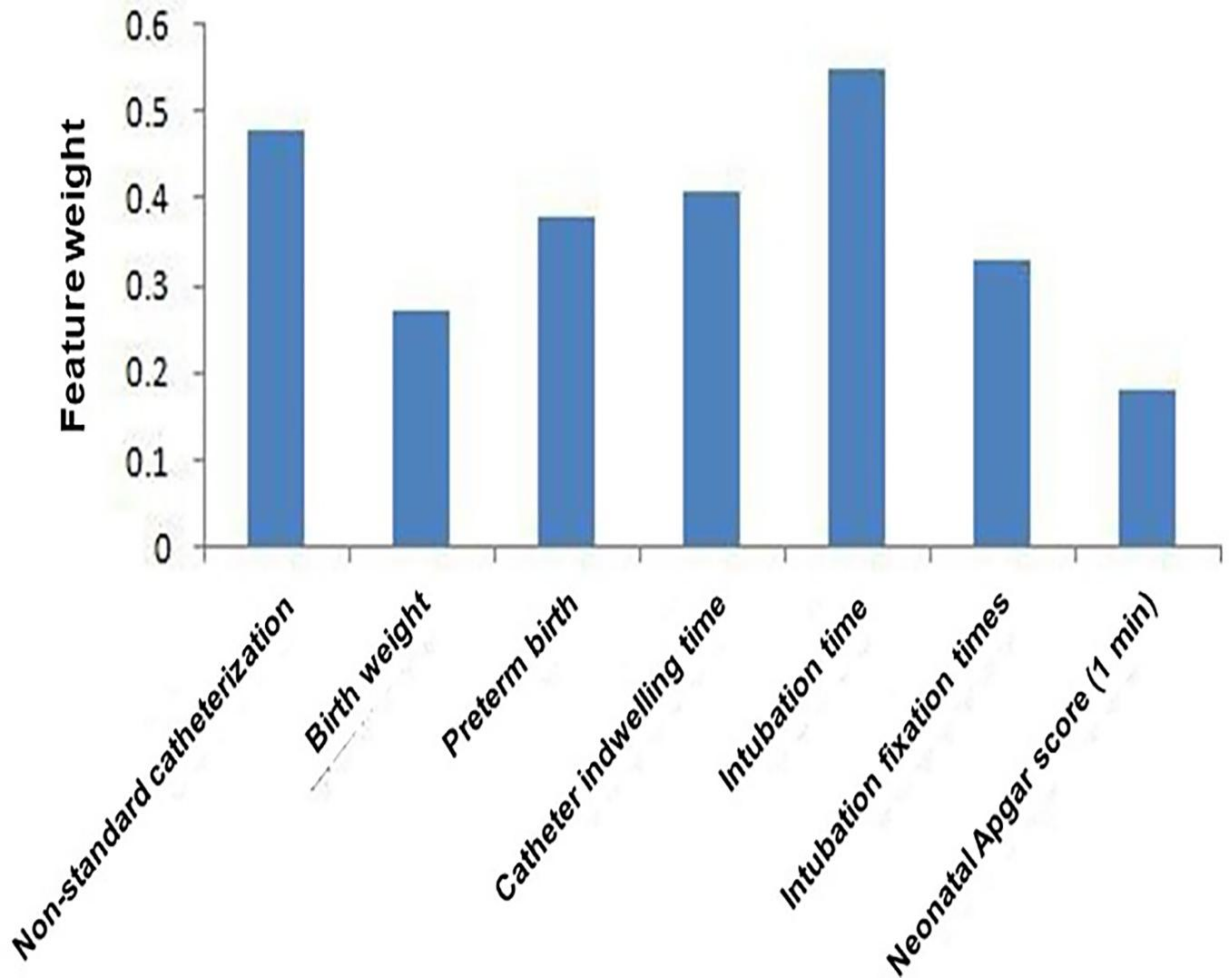
Multivariate logistic regression analysis of factors associated with complications of umbilical venous catheterization. This figure presents the results of the multivariate logistic regression model identifying independent factors associated with UVC-related complications. Odds ratios (ORs) with 95% confidence intervals are displayed for each variable, illustrating risk factors (OR > 1) and protective factors (OR < 1) after adjustment for potential confounders.

The multivariable analysis indicated that the independent risk factors for UVC-associated complications were non-standard catheterization (OR 5.24, 95% CI 2.15–12.78), longer indwelling time (>8 days; OR 4.50, 95% CI 1.95–10.38), preterm birth (OR 3.12, 95% CI 1.45–6.72), lower birth weight (OR 2.45, 95% CI 1.10–5.45), and a lower 1 min Apgar score (OR 1.85, 95% CI 1.05–3.25). Factors protective against UVC-related complications included a longer time for catheterization (OR 0.82, 95% CI 0.70–0.95) and fewer attempts at fixation (OR 0.65, 95% CI 0.45–0.93). An odds ratio of less than one indicates a protective association, rather than an increased risk (Figure 2).

### 3.5 XGBoost model performance and feature importance

**Figure 3:**
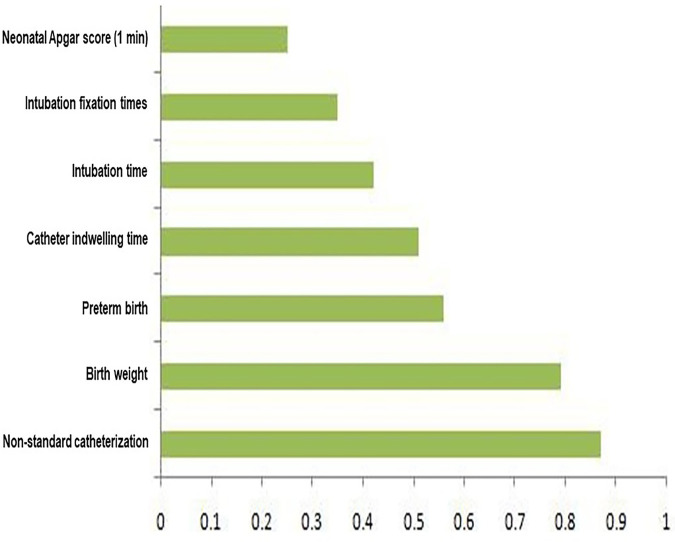
Construction of the XGBoost-based prediction model for UVC-related complications. This figure illustrates the development of the XGBoost prediction model using clinically relevant variables to estimate the probability of complications following umbilical venous catheterization. Feature importance weights reflect each variable's relative contribution to the model's predictive output. Ventilator support and inotrope use were included as adjustment variables in the predictive model, but their relative feature importance was lower compared to procedural and neonatal factors (e.g., non-standard catheterization, birth weight, preterm birth).

Feature importance analysis identified the following variables as key contributors to the model's prediction of UVC-related complications: catheterization insertion time, non-standard catheterization, indwelling catheter time, preterm birth, number of fixation attempts, birth weight, and 1 min Apgar score (Figure 3). Although ventilator support and inotrope use were included in the model, their SHAP values were low, indicating that these factors had limited influence on predicted complication risk compared to procedural and neonatal characteristics.

**Figure 4:**
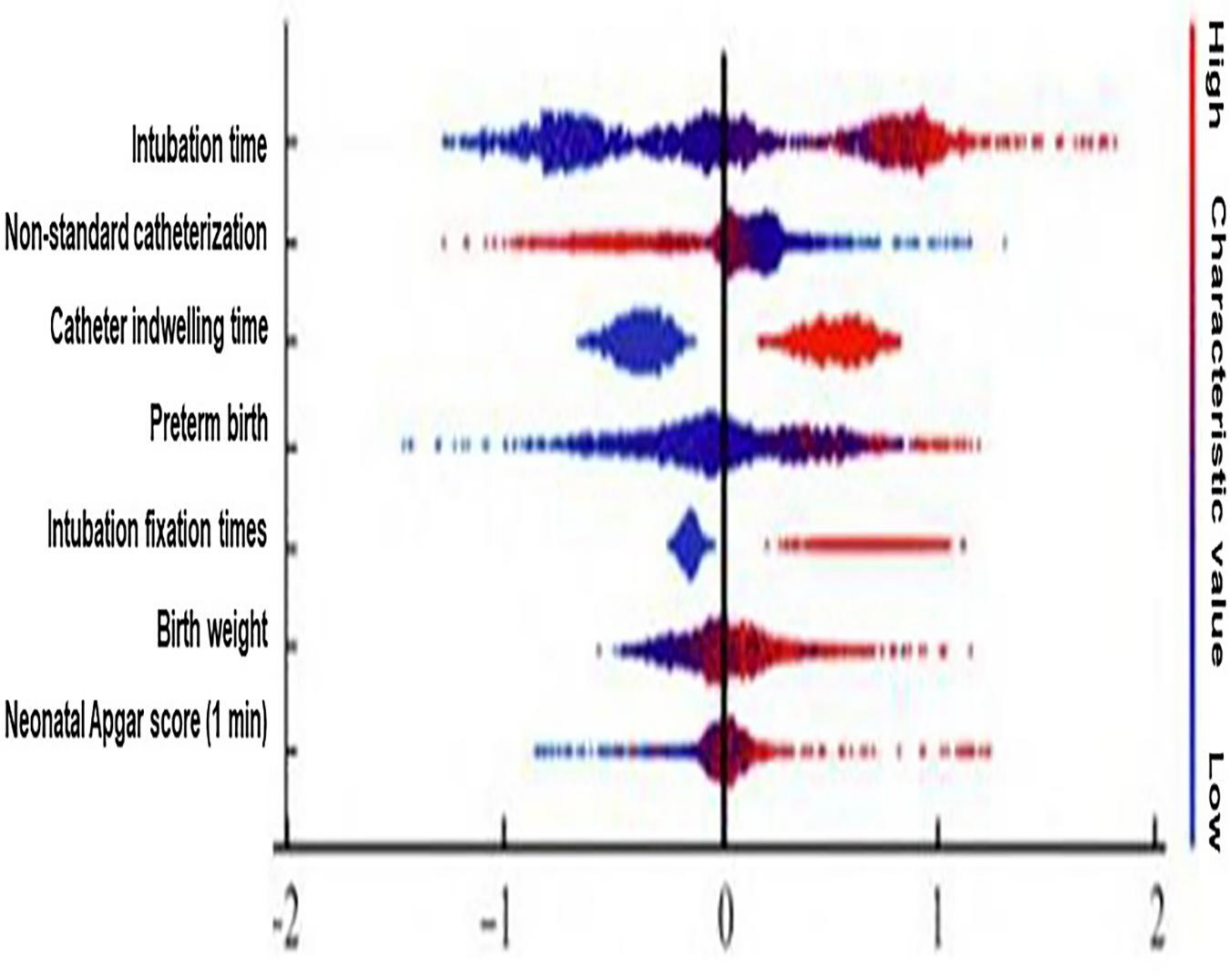
Performance evaluation of the XGBoost prediction model. This figure shows the performance of the XGBoost model using receiver operating characteristic (ROC) analysis, calibration curves, and decision curve analysis. These evaluations demonstrate the model's discrimination ability, agreement between predicted and observed risks, and potential clinical net benefit across a range of threshold probabilities.

Calibration analysis demonstrated good agreement between the predicted probabilities of complications from the XGBoost model and the observed outcomes. Decision curve analysis further revealed a net clinical benefit when using the model across a range of threshold probabilities (Figure 4).

Although ventilator support and inotrope use were included in the model, their SHAP values were low, indicating limited influence on predicted complication risk compared to procedural and neonatal characteristics.

### 3.6 SHAP interpretation of the XGBoost model

**Figure 5:**
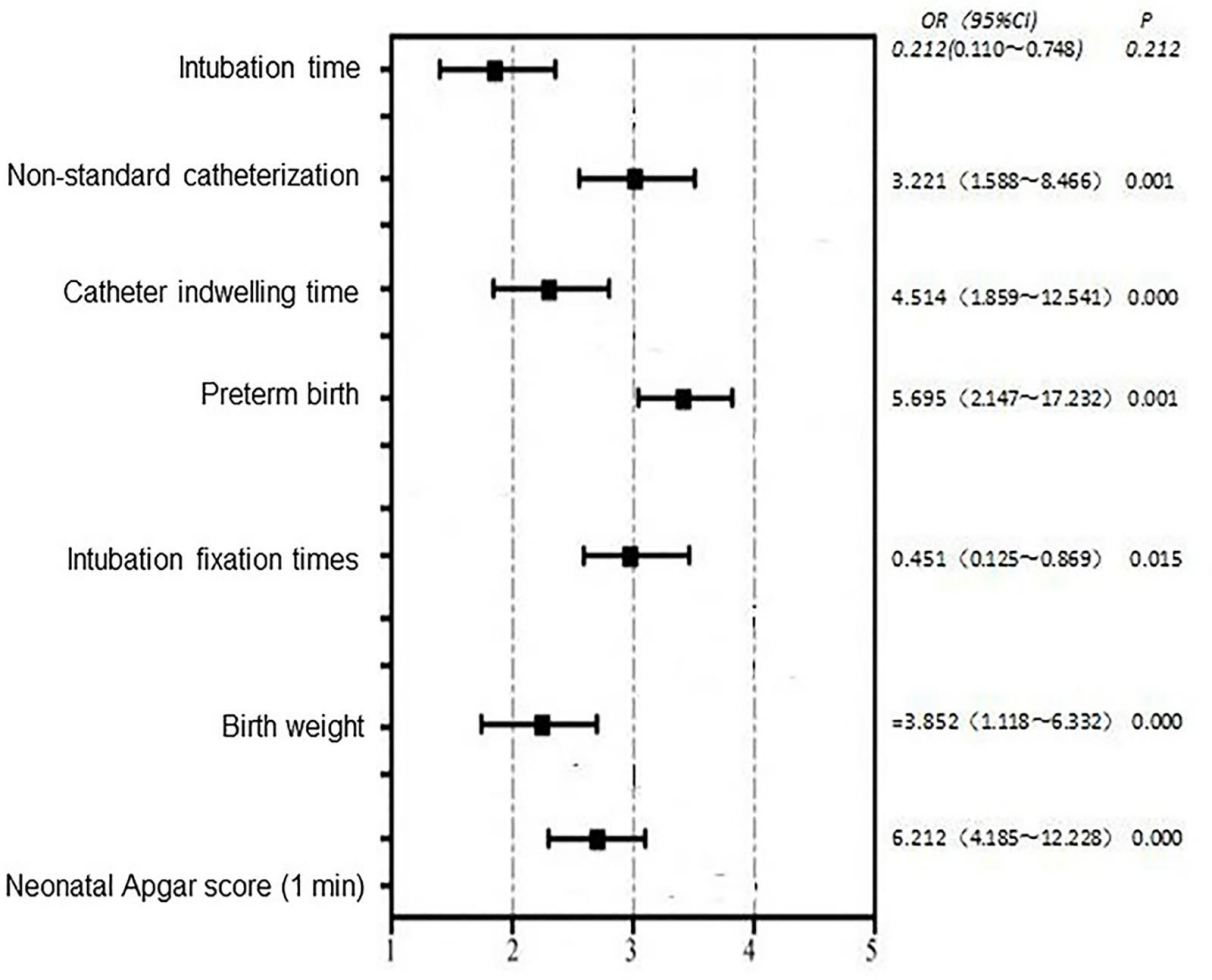
Feature importance ranking based on SHAP analysis. This figure displays the global feature ranking importance derived from Shapley Additive exPlanations (SHAP), highlighting the variables with the greatest overall influence on the XGBoost model's predictions of UVC-related complications.

**Figure 6:**
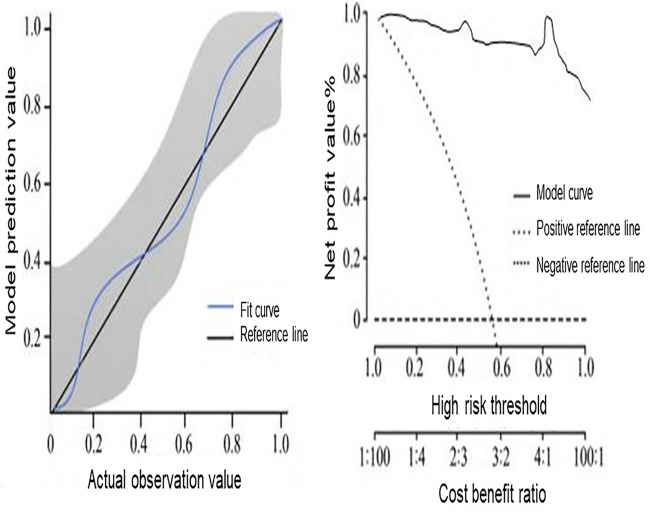
SHAP summary scatter plot of variables influencing UVC-related complication risk. This scatter plot visualizes the distribution of SHAP values for individual predictors across all observations. Each point represents a neonate, with color indicating the magnitude of the feature value. Higher SHAP values correspond to increased predicted risk of UVC-related complications, whereas lower values indicate protective effects.

SHAP analysis enhanced the interpretability of the XGBoost model by quantifying the contribution of each predictor to individual risk estimates. The variables with the greatest influence on predicted complication risk were non-standard catheterization (the highest absolute SHAP value, 0.87), infant birth weight (SHAP value = 0.79), preterm birth (SHAP value = 0.65), and catheter indwelling time (SHAP value = 0.51). Higher SHAP values were consistently associated with an increased probability of UVC-related complications, while lower values indicated protective effects (Figures 5, 6).

## 4 Discussion

UVC plays a vital role in the emergency management and early nutritional support of premature and critically ill newborns. This is largely attributable to the umbilical vein's large lumen, superficial location, and high first-time insertion success rate (5). However, despite these important clinical benefits, UVC is associated with both procedure- and catheter-related complications—particularly when the catheter tip is malpositioned or when post-insertion monitoring is inadequate (6, 7).

In the present cohort, UVC-related complications were limited in scope and severity. Observed complications included catheter malposition or early dislodgement, portal or intrahepatic pneumatosis occurring shortly after insertion, blood reflux related to catheter positioning, and local umbilical findings such as redness, swelling, or exudate. Importantly, none of the severe complications reported in other studies—such as cardiac tamponade, clinically significant arrhythmias, or catheter-related bloodstream infections—were observed in this series. Nevertheless, with a sample size of 132 neonates, this study may be underpowered to draw definitive conclusions regarding the true incidence of these rare but serious events under ultrasound guidance. As such, this distinction is important to avoid overinterpretation and to contextualize our findings within the broader literature (8–10). While our cohort provides a robust basis for identifying common clinical risk factors in real-world settings, it does not permit reliable estimation of rare adverse event rates.

The key advantage of real-time bedside ultrasound in this study lies in its ability to visualize catheter advancement and tip placement during insertion, enabling immediate correction of malposition and reducing the need for repeated post-insertion radiographs. Traditional X-ray verification, by contrast, offers limited accuracy and often requires multiple exposures, thereby increasing radiation exposure in this vulnerable population (10–12). Ultrasound has also been shown to correctly identify catheter tips positioned within the atrium, even when X-ray findings appear normal—particularly in very low birth weight infants (13, 14). Although this study did not quantitatively assess radiation reduction, the use of ultrasound as the primary verification method eliminated routine radiographic exposure, supporting the growing body of evidence that ultrasound improves accuracy and minimizes radiation compared to conventional X-ray confirmation.

The existing literature documents a wide range of UVC-associated complications, including pericardial effusion (cardiac tamponade), pleural effusion, arrhythmias, retained catheters, and bloodstream infections (15–19). While none of these severe events were observed in our cohort, their absence should be interpreted with caution given the sample size. Large cohort studies have demonstrated a significant association between catheter dislodgement-related adverse events and longer catheter dwell times, underscoring the importance of ongoing position monitoring until removal (20, 21). Furthermore, serial ultrasound assessments may facilitate early detection of malpositioned catheters, potentially preventing life-threatening complications such as hepatic injury resulting from catheter tips positioned below the diaphragm (22, 23).

Our findings are largely consistent with those of previously reported studies. Prolonged catheter indwelling (>8 days), non-standard catheterization technique, preterm birth, low birth weight, and lower 1-minute Apgar scores were all identified as major risk factors for UVC-related complications in premature infants. Conversely, shorter time from birth to catheterization and fewer fixation attempts were associated with a trend toward fewer complications.

Notably, the relationship between birth weight and UVC complications remains debated in the literature, with conflicting findings reported across studies (24–27). Our data support the hypothesis that very low birth weight premature infants may be particularly susceptible to localized inflammatory or exudative responses, likely due to greater tissue fragility and reduced physiological reserve.

To support clinical decision-making, we developed a predictive model using the XGBoost algorithm and applied SHAP analysis to enhance interpretability. This model may assist neonatologists in identifying premature, low-birth-weight infants at increased risk for UVC-related complications—those who might benefit from more frequent ultrasound monitoring of catheter position or shorter dwell times. SHAP analysis identified four factors with the greatest impact on predicted risk: (1) non-standard catheterization technique, (2) birth weight, (3) preterm birth, and (4) catheter indwelling time. Together, these findings underscore the importance of sound procedural technique and high-quality post-insertion care.

This study has several limitations. First, the absence of a synchronous control group (e.g., traditional X-ray guided UVC) precludes direct statistical comparison of efficacy between ultrasound-guided and conventional methods. Although we adjusted for key clinical confounders such as ventilator support and inotrope use, other unmeasured factors—including variations in neonatologist expertise or the urgency of emergency insertions—may still influence the results. As such, the observed improvements are inferred through comparisons with historical or external literature.

Second, the relatively small number of complications (*n* = 20) and the two-week follow-up period may limit the precision of risk estimates and the stability of the predictive model. The sample size is also likely insufficient to capture rare, high-severity adverse events, underscoring the need for validation in larger, multi-center prospective cohorts. Moreover, the short observation window may have led to underdetection of late-onset complications, such as catheter-related bloodstream infections (CRBSI), delayed thrombosis, or hepatic injury resulting from prior malposition. However, informal reviews of clinical follow-up records and discharge summaries for this cohort revealed no documented cases of late-onset CRBSI or significant delayed sequelae beyond the two-week period, suggesting that the study timeframe captured the vast majority of clinically relevant events.

Although our internally validated model demonstrates strong predictive performance, it has yet to be externally validated for routine clinical application. Furthermore, the generalizability of our findings may be limited, as successful ultrasound-guided UVC insertion depends on the availability of high-frequency linear transducers and clinicians with specialized training in POCUS—resources that may not be uniformly available across all neonatal centers.

In summary, we have demonstrated that ultrasound-guided UVC placement in neonates is a safe and accurate procedure. While radiation reduction was not directly quantified in this sample, the implementation of an ultrasound-first protocol supports a clinical workflow that minimizes radiation exposure in this vulnerable population.

## 5 Conclusion

Bedside real-time ultrasound for UVC placement in newborns is a safe and accurate procedure that allows clinicians to visualize and adjust the catheter tip position during insertion. Although further randomized controlled trials are needed to definitively establish its superiority over X-ray guidance, our findings support its clinical utility. The XGBoost-based prediction model, combined with SHAP interpretation, demonstrates potential for reliably estimating the risk of UVC-related complications. However, given variability in equipment and operator expertise across institutions, external validation is necessary before widespread clinical implementation.

## Data Availability

The original contributions presented in the study are included in the article/**Supplementary Material**, further inquiries can be directed to the corresponding author.

## Supplementary material

Supplementary Figure S1 Representative ultrasound images of the bedside real-time ultrasound-guided UVC insertion technique. **(A)**: Transverse view showing the initial entry and orientation of the catheter within the umbilical vein. **(B)**: Longitudinal view illustrating the advancement of the catheter tip toward the junction of the inferior vena cava and the right atrium.
