# Supplementary figures and images for: Application of bedside real-time ultrasound–guided umbilical venous catheterization in neonates and development of an XGBoost-based complication prediction model: a retrospective study
Source: Front Pediatr. 2026 May 21;14:1797779. doi: 10.3389/fped.2026.1797779 (PMC13234701; doi:10.3389/fped.2026.1797779)

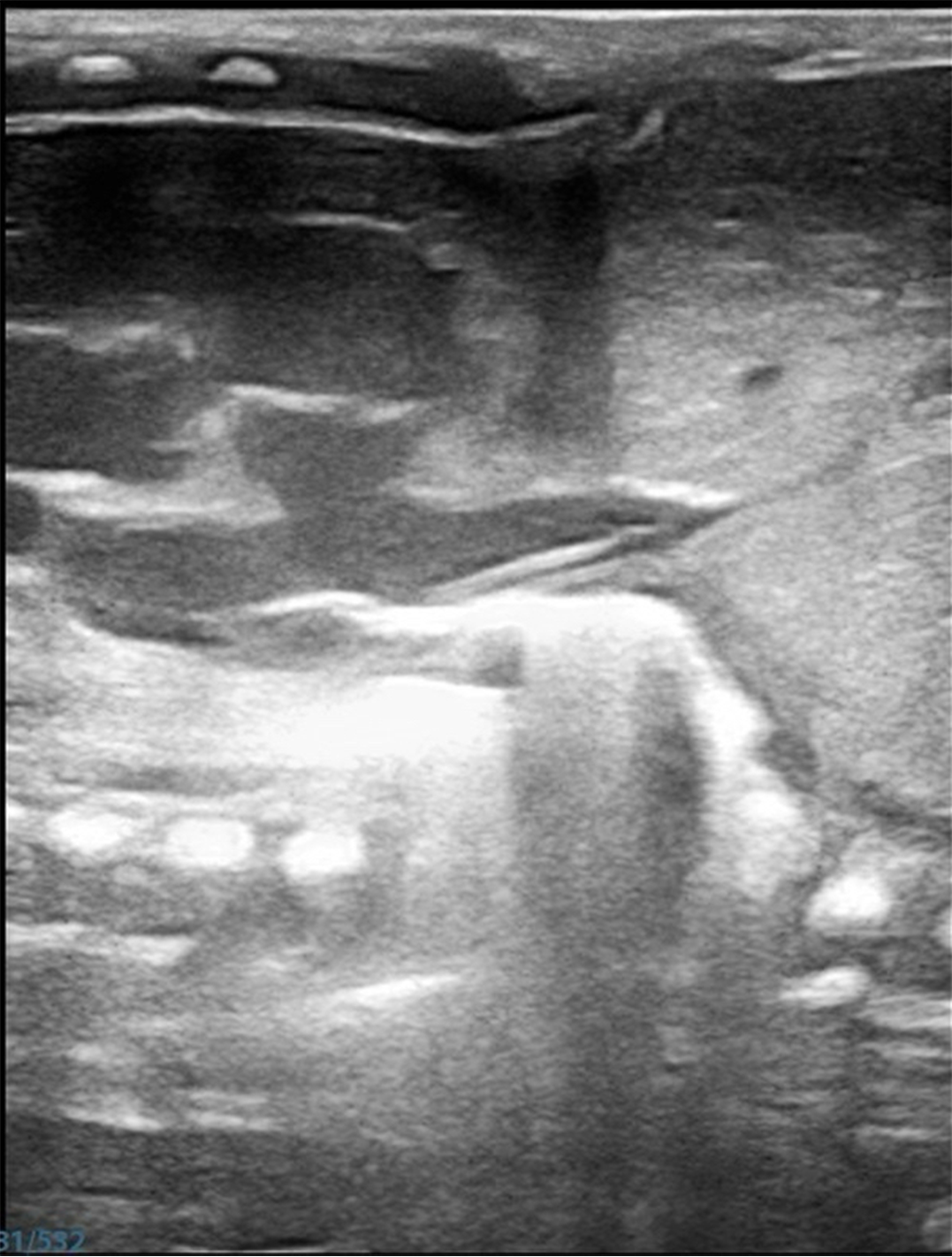

Supplement: Supplementary Figure S1 — Representative ultrasound images of the bedside real-time ultrasound-guided UVC insertion technique. (A): Transverse view showing the initial entry and orientation of the catheter within the umbilical vein. (B): Longitudinal view illustrating the advancement of the catheter tip toward the junction of the inferior vena cava and the right atrium. [file Image1.tif]
